# Beyond the Exome: The Role of Noncoding and Regulatory Variants in Monogenic Diseases

**DOI:** 10.3390/cimb47121038

**Published:** 2025-12-12

**Authors:** Efthalia Moustakli, Nektaria Zagorianakou, Stylianos Makrydimas, Andreas Miltiadous, Alexandros T. Tzallas, George Makrydimas

**Affiliations:** 1 Department of Nursing, School of Health Sciences, University of Ioannina, 4th Kilometer National Highway Str. Ioannina-Athens, 45500 Ioannina, Greece; ef.moustakli@uoi.gr (E.M.); zagorianakou@uoi.gr (N.Z.); 2 Human Computer Interaction Laboratory, Department of Informatics and Telecommunications, University of Ioannina, Kostakioi, 47150 Arta, Greece; a.miltiadous@uoi.gr (A.M.); tzallas@uoi.gr (A.T.T.); 3 Medical School, Aristotle University of Thessaloniki, 54124 Thessaloniki, Greece; smakrydimas@gmail.com; 4 Department of Obstetrics & Gynecology, University Hospital of Ioannina, 45500 Ioannina, Greece

**Keywords:** noncoding variants, monogenic disease, whole-genome sequencing, splicing, regulatory mutations, precision medicine

## Abstract

Analysis of coding areas has long been used to study monogenic illnesses, but despite the extensive use of whole-exome sequencing (WES), up to half of suspected cases remain genetically unexplained. Variants outside coding areas can alter splicing, transcript stability, or gene regulation, compromising normal gene activity. These include mutations in noncoding RNAs, promoters, enhancers, deep intronic sequences, and untranslated regions (UTRs). Several well-known disorders have been linked to these mechanisms, including β-thalassemia caused by deep intronic mutations leading to aberrant splicing, familial hypercholesterolemia caused by promoter defects affecting LDLR expression, and inherited retinal diseases driven by noncoding variants influencing retinal gene regulation. These instances show that pathogenic variation is not limited to the exome and can have significant clinical implications. This review summarizes current understanding of noncoding and regulatory variants in monogenic diseases, discusses how they influence diagnosis and therapy, and highlights integrative approaches combining genomic, transcriptomic, and epigenomic data. Multi-layered research has increased diagnostic accuracy and unveiled new therapeutic potentials, although noncoding variations make the connection between genotype and phenotype more complex. Noncoding regions will need to be incorporated into standard diagnostic procedures to convert molecular insights into concrete therapeutic applications in the future. Predictive algorithms, patient-derived model systems, and functional validation testing will all help to simplify this process.

## 1. Introduction

Monogenic diseases, resulting from pathogenic mutations in a single gene, have historically been regarded as the most fundamental classifications of human genetic disorders. The old paradigm of “one gene–one phenotype” provided a strong basis for understanding disease pathophysiology and fueled decades of successful gene discovery studies [1]. Next-generation sequencing (NGS) technologies, especially WES, have revolutionized clinical genetics by enabling rapid, low-cost, and comprehensive analysis of the coding regions that harbor the majority of known disease-causing mutations. WES has become a primary diagnostic tool in many clinical settings, greatly increasing diagnostic yields and lowering the so-called “diagnostic odyssey” for patients with rare diseases [2,3].

However, up to 40–50% of individuals who have a high clinical suspicion of a monogenic disease cannot be identified by exome analysis, even with the widespread use of WES. This “diagnostic gap” has led to a greater understanding of the pathophysiology of disease, revealing that detrimental variants may occur beyond the exome and that noncoding and regulatory components are more relevant than previously believed [4]. These elements play a critical role in governing the spatial, temporal, and mechanistic regulation of gene expression. Precise regulation of gene expression in terms of spatial, temporal, and mechanistic aspects depends on genomic elements such as deep intronic sequences, promoters, enhancers, silencers, and UTRs [5,6]. Long noncoding RNAs (lncRNAs) and microRNAs (miRNAs) represent two classes of functional noncoding RNAs whose discovery is progressively expanding. This adds another level of regulatory complexity that may be able to alter disease indicators [7].

According to recent studies, alterations in these noncoding elements can be pathogenic. The CFTR, DMD, and PAH genes demonstrate how deep intronic changes that enable novel splice donor or acceptor sites can lead to the generation of shorter or defective proteins [8,9,10]. Transcript stability or translation efficiency may be affected by variations in the 5′ and 3′ UTRs that interact with regulatory secondary structures or microRNA binding motifs. Inadequate penetrance and phenotypic heterogeneity may arise from mutations in lncRNAs or other regulatory RNAs that disrupt the coordinated control of entire gene networks [11].

Whole genome sequencing (WGS) and integrated multi-omics techniques have expanded the variety of mutations found in monogenic disorders, highlighting the importance of searching “beyond the exome.” However, the lack of functional annotations of regulatory areas and the development of computational techniques make it difficult to predict the functional effects of noncoding variations [12,13]. Identifying these pathways is expected to significantly impact both basic research and clinical practice. Furthermore, these findings pave the way for new therapeutic approaches, including as RNA editing, CRISPR-based enhancer targeting, and splice-switching antisense oligonucleotides (ASOs), to rectify harmful regulatory changes [14].

Although several reviews have addressed the limitations of exome-focused diagnostics, there remains a need for a comprehensive synthesis that integrates emerging categories of noncoding pathogenic variants with the rapidly advancing function-al-genomics toolkit [15]. Recent developments, such as high-resolution WGS, long-read sequencing, CRISPR-based regulatory interrogation, and multi-omics strategies, have significantly enhanced the identification and validation of regulatory variants [16,17]. This review aims to address this gap by presenting an updated, mechanistically focused framework for understanding the contribution of noncoding mutations to monogenic disease and by demonstrating how these insights improved diagnostic practice and novel therapeutic opportunities.

This review is intended for clinical geneticists, molecular diagnosticians, and re-searchers who develop functional or computational approaches for variant interpretation. It summarizes current knowledge, integrates emerging methodologies, and pro-vides illustrative disease examples to serve as a practical and up-to-date guide for ad-dressing unsolved monogenic cases that remain after WES.

The significance of noncoding and regulatory variants in the molecular pathogenesis of monogenic disorders is examined in this article. First, we classify mutations based on their mode of action and chromosomal location, and we discover commonalities across a wide range of disease scenarios. The computational and experimental approaches for identifying and functionally validating noncoding variants are then discussed, including transcriptome analysis, WGS, and CRISPR-based functional genomics. We highlight well-characterized variants associated with representative monogenic illnesses, including cystic fibrosis, Duchenne muscular dystrophy, β-thalassemia, familial hypercholesterolemia, and hereditary retinal diseases, to demonstrate these ideas. We highlight the practical implications at the end, including the potential for precision medicine, increased diagnostic output, and targeted treatment development. Collectively, these instances highlight the therapeutic importance of searching “beyond the exome” and show how mechanistic discoveries have influenced better diagnostics and, occasionally, therapeutic approaches.

## 2. Methods

This study synthesizes the existing literature on the function of regulatory and noncoding variations in monogenic diseases using a narrative method. Relevant articles were found by using keyword combinations such as “noncoding variants,” “regulatory mutations,” “monogenic disorders,” “whole-genome sequencing,” and “functional validation” in searches of PubMed, Scopus, and Web of Science. Priority was given to the literature released between 2000 and 2025, with an emphasis on peer-reviewed original research, reviews, and genetic studies with a clinical focus.

In monogenic conditions, articles that described noncoding or regulatory variations with proven or suggested harmful effects were included, especially those that were clarified by transcriptomic, genomic, or multi-omics investigations. Excluded were studies that focused only on complicated disorders or polygenic characteristics. Additional references were found using the bibliographies of important articles and reputable databases such as ClinVar, gnomAD, and GTEx.

The information was organized thematically, categorizing changes by genomic location (promoter/enhancer, deep intronic, untranslated regions, and noncoding RNAs) and molecular mechanism (splicing disruption, transcriptional dysregulation, altered RNA stability). Several well-characterized illnesses, including inherited retinal diseases, Duchenne muscular dystrophy, cystic fibrosis, β-thalassemia, and familial hypercholesterolemia, were used as illustrative examples to highlight mechanistic and clinical relevance.

## 3. Categories of Noncoding and Regulatory Variants

**Figure 1:**
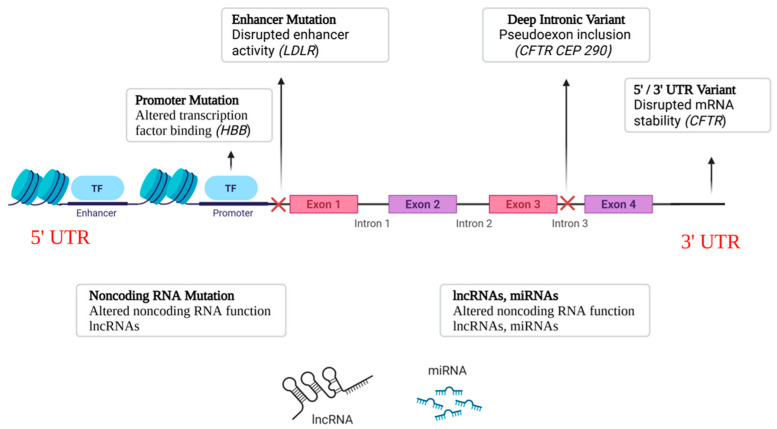
Classes of Noncoding Variants. Schematic representation of where major noncoding variants occur relative to a gene. HBB: β-globin; LDLR: low-density lipoprotein receptor; CFTR: cystic fibrosis transmembrane conductance regulator; CEP290: centrosomal protein 290.

Noncoding and regulatory variants can affect gene activity through a variety of methods, in addition to modifications to the protein-coding sequence. These variations are usually found in genes that encode regulatory RNAs, introns, UTRs, enhancers, and promoters. Their impacts vary from changing RNA stability and translation to changing transcriptional activity and splicing. The primary categories of noncoding variations linked to the pathophysiology of monogenic diseases are outlined below [18,19,20]. An overview of the main classes of noncoding variants and their genomic positions is shown in Figure 1.

### 3.1. Promoter and Enhancer Variants

The cis-regulatory components known as promoters and enhancers regulate the spatiotemporal expression of genes. Reduced or ectopic gene expression may result from promoter variations that alter transcription factor binding motifs or key promoter elements (such as the TATA box and transcription start sites). By altering chromatin accessibility or hindering the recruitment of transcriptional activators, enhancer mutations can also decrease the transcription of target genes [21,22,23].

Several monogenic illnesses have well-documented promoter variations. For example, β-thalassemia is known to be caused by HBB promoter mutations, including −28A>G and −29A>G, which result in decreased β-globin production and a more severe form of anemia [24]. Variants in the LDLR promoter prevent Sp1 transcription factor binding in familial hypercholesterolemia, lowering the expression of the LDL receptor and increasing plasma LDL cholesterol levels. Enhancer variants regulating SOX10 and PAX6 have also been discovered in recent WGS investigations, which help to explain previously unresolved cases of Waardenburg syndrome and aniridia, respectively [25,26].

### 3.2. Deep Intronic Variants

Deep intronic variations can trigger cryptic splice sites or produce unique splice motifs because they are situated distant from canonical splice donor or acceptor sites. These modifications could lead to frameshifts or premature termination codons by causing pseudoexon inclusion, partial intron retention, or exon skipping [27,28].

A typical example is the CFTR variation c.3718-2477C>T (3849+10kbC>T), which produces a nonfunctional CFTR protein by introducing a cryptic splicing site that results in pseudoexon inclusion [29]. Similarly, the c.2991+1655A>G mutation forms a strong donor splice site in CEP290-associated Leber congenital amaurosis, resulting in faulty mRNA splicing and retinal degeneration. In addition to increasing diagnostic yield, these findings have sparked the development of therapeutics. ASOs have been developed to restore normal splicing in both CFTR and CEP290 abnormalities by covering cryptic splice sites [30,31].

### 3.3. 5′ and 3′ UTR Variants

UTRs are crucial for controlling the localization, translation efficiency, and stability of mRNA. Variants in the 5′ UTR may interfere with secondary structures or upstream open reading frames (uORFs) that affect translation start and ribosome scanning. RNA-binding protein or microRNA binding sites are often impacted by 3′ UTR variations, which can change the half-life of transcripts [32].

For instance, pathogenic 5′ UTR mutations in FMR1 can cause hypermethylation and gene silencing, resulting in fragile X syndrome. It has been demonstrated that 3′ UTR variations, which mimic loss-of-function coding mutations, destabilize mRNA transcripts and reduce protein production in immune deficiency conditions caused by GATA2 haploinsufficiency [33].

### 3.4. Noncoding RNA Mutations

Gene expression networks are fine-tuned in part by LncRNAs, miRNAs, and other short RNAs. Multiple target genes may thus be affected in a cascade by mutations that alter their expression or function [34,35].

Several instances highlight their significance, although they are still little understood. Familial melanoma risk has been linked to mutations in the lncRNA ANRIL, which controls the CDKN2A/B locus [36]. Similarly, cancer susceptibility, neurodevelopmental disorders, and congenital heart problems have all been connected to mutations that impact miRNA seed regions or miRNA-binding sites. To diagnose monogenic diseases, our results highlight the necessity of a thorough evaluation of regulatory RNA genes [37]. Table 1 outlines the main categories of noncoding and regulatory variants associated with monogenic diseases.

Another significant type of noncoding RNAs associated with monogenic diseases is circular RNAs (circRNAs). CircRNAs, which are produced by back-splicing, provide highly stable covalently closed structures. Many circRNAs serve as scaffolds for RNA-binding proteins, transcription regulators, or miRNA sponges.

CircRNAs play a crucial role in the competitive endogenous RNA (ceRNA) network, where mRNAs, lncRNAs, and circRNAs vie for common miRNA binding sites. This process allows changes in circRNA abundance to indirectly control the expression of genes that code for proteins. Dysregulated circRNAs have been linked to muscular dystrophies, neurodevelopmental syndromes, and hematologic disorders, highlighting their potential pathogenic and diagnostic implications, even though research in monogenic disease is still in its early stages.

## 4. Methods for Detection and Functional Validation

In contrast to exonic mutations, which typically alter protein sequences directly, noncoding and regulatory variants are more challenging to detect and interpret because their effects on gene expression, splicing, or RNA stability are often subtle and context-dependent [18,41]. Identifying and characterizing regulatory elements usually requires a mix of transcriptomic, genomic, experimental, and computational methods. While CRISPR-based assays can directly evaluate the functional impact of a regulatory variation, RNA-seq can show how it changes gene expression. Each of these methods provides a distinct layer of understanding [42]. Utilizing a variety of approaches enhances the capacity to rank the variations that are most likely to cause disease and validates experimental results. Researchers can gain a deeper understanding of how regulatory changes impact biological pathways, which in turn influence observable traits or disease phenotypes, by combining multiple datasets [43].

### 4.1. Next-Generation Sequencing Approaches

WES has long served as the primary method for identifying pathogenic variants in monogenic disorders. By focusing on the protein-coding regions, which represent only 1–2% of the genome, WES combines a well-established analytical workflow with proven clinical utility and relatively low cost, making it a practical and widely adopted diagnostic tool [44]. Although promoters, enhancers, UTRs, and deep introns are examples of noncoding or regulatory areas that WES does not analyze, these regions may show alterations linked to disease. Therefore, reliance on WES may lead to the loss of modifications that affect gene regulation, splicing, or chromatin organization [45].

Due to its ability to include both coding and noncoding regions, WGS offers a more complete evaluation. Large indels, copy-number changes, deep intronic modifications, and single-nucleotide and structural variations (SVs) that could impair gene regulation can all be found using WGS [46]. This increased coverage increases the diagnostic yield, especially for conditions where splicing, gene expression, or three-dimensional chromatin architecture are altered by noncoding or structural alterations. Interpreting noncoding variation is problematic due to the vast amount of variants and poor functional annotation [15].

Advances in sequencing technologies have led to a major growth in WGS applications. Accurate haplotype phasing, the discovery of complex structural variants in repetitive or GC-rich areas, and direct measurement of DNA methylation, providing insights into epigenetic control, are all made possible by long-read platforms (e.g., Oxford Nanopore and PacBio HiFi) [47,48]. A more accurate genomic representation is achieved using hybrid techniques, which combine short-read accuracy with long-read structural insights. Large genomic rearrangements, repeat expansions, and segmental duplications that affect gene regulation can be better detected using complementary approaches like optical mapping and linked-read sequencing (10x Genomics and Bionano Genomics) [49].

To connect noncoding changes to functional outcomes, it is becoming important to integrate transcriptome and epigenomic reference maps, such as those in ENCODE, Roadmap Epigenomics, and GTEx, with WGS data. With success rates ranging from roughly 40% to 60%, WGS frequently offers a greater diagnostic yield than WES in rare disease research [50]. Important pathogenic mutations might be located outside of conventional coding areas, as evidenced by the fact that many patients still require a definitive molecular diagnosis. These results underline the necessity of integrated approaches that better assess the functional impact of noncoding variation by combining computational modeling, transcriptome profiling, and experimental assays [51].

### 4.2. Transcriptomic and Functional Genomic Approaches

RNA sequencing (RNA-seq) is a powerful tool for uncovering how noncoding and regulatory variants influence gene expression. Unlike DNA-based approaches, RNA-seq offers a direct snapshot of transcription, revealing the impact of specific mutations on splicing, transcript stability, and global gene expression [52]. Deep intronic variants, for example, may disrupt normal gene expression through allele-specific effects, aberrant splicing, or pseudoexon inclusion—mutations that often escape detection by conventional exome or genome sequencing approaches [53].

Applying RNA-seq to disease-relevant tissues or patient-derived models, including fibroblasts, lymphoblastoid cell lines, or organoids derived from induced pluripotent stem cells, further increases its usefulness [54]. These platforms give researchers a more physiologically relevant viewpoint by enabling them to assess variation effects within tissue-specific transcriptional and splicing systems. Oxford Nanopore and PacBio Iso-Seq are two recent developments in long-read RNA sequencing technology that have made it possible to discover complicated isoforms, precisely phase transcripts, and thoroughly analyze full-length splice variants [55].

It is possible to correlate noncoding mutations with unbalanced expression or changed gene dosage by combining RNA-seq data with DNA variant data. By associating transcriptional disruptions with particular sequence changes, quantitative transcriptome analysis and allele-specific expression studies also help differentiate benign from harmful variations [56]. Confirming anticipated transcriptome changes still requires functional confirmation. While dual-luciferase and fluorescent reporter assays offer extra measurements of transcript abundance and splicing efficiency, in vitro minigene reporter assays provide a flexible method for investigating splicing outcomes, such as exon skipping, pseudoexon incorporation, and cryptic splice site usage [57].

Traditional techniques, including RT-PCR, qPCR, and Northern blotting, are commonly employed to validate RNA-seq findings. Single-cell RNA sequencing (scRNA-seq) further enables the assessment of regulatory variant effects across specific cell types [58,59]. Researchers can establish a more direct link between specific DNA variations and their biological effects by combining transcriptome data with functional genomic analysis. In diseases like inherited retinal diseases and muscular dystrophy, this type of comprehensive method has previously categorized how specific noncoding mutations change gene regulation [60]. Beyond increasing the precision of diagnosis, these also aid in the discovery of novel treatment prospects, such as by identifying targets for gene editing or RNA-based treatments [61].

### 4.3. Computational Prediction and Annotation

Computational prediction frameworks play a critical role in selecting noncoding variants for functional follow-up, particularly in light of the extensive variant burden revealed by WGS. These methods provide a thorough assessment of the possible functional impact of a variant by combining numerous features, such as chromatin accessibility, transcription factor binding, RNA splicing cues, and sequence conservation [62]. Multiple genomic annotations are combined by frameworks such as DeepSEA, EIGEN, and CADD to evaluate the possible clinical relevance of noncoding variations. Highly accurate identification of cryptic splice site alterations linked to monogenic disorders has been shown by specialized techniques such as SpliceAI, MMSplice, and SQUIRLS. They can predict the effects of intronic mutations on RNA splicing with accuracy [63].

These predictive models now perform noticeably better because to developments in deep learning. Convolutional neural networks trained on large-scale epigenomic datasets (such as ENCODE and Roadmap Epigenomics) are used by models like Basenji, Enformer, and DeepSplice to forecast the effects of single-nucleotide variations on transcription and chromatin structure in various cell types. These AI-driven frameworks that capture long-range enhancer–promoter interactions help to better understand the impact of distal regulatory variants [64].

Accurate variant interpretation also requires the inclusion of functional and population-level data. Allele frequencies, tissue-specific regulatory effects, and eQTL connections are available through databases such as gnomAD, ClinVar, GTEx, and RegulomeDB [65]. It is possible to identify variants that are likely to have functional impacts while weeding out those that are less important by combining computational predictions with experimental annotations [66].

However, computational predictions are still influenced by biological context and carry an inherent degree of uncertainty. Many models fail to capture tissue-specific or developmental effects due to incomplete annotation of regulatory elements [67]. Therefore, these tools are most effective as prioritization aids rather than definitive classifiers. The most reliable approach to interpret noncoding variations in monogenic disorders remains integrating computational predictions with experimental validation and multi-omics datasets [45].

### 4.4. Integrated Multi-Omics Approaches

Interpreting noncoding and regulatory variations requires integrating genomic, transcriptomic, and epigenomic data more and more since it allows researchers to link DNA alterations to functional outcomes [20]. Systems-level methods that combine RNAseq, WGS, and epigenomic profiling can show how variations affect chromatin architecture, gene expression, and splicing. High-resolution mapping of these regulatory effects is aided by experimental methods including ATAC-seq (DNA accessibility), Hi-C and Capture-C (chromatin conformation), and ChIP-seq (histone alterations) [68].

The investigation of regulatory variance across many cell types is made possible by single-cell multi-omics technologies, such as scRNA-seq, scATAC-seq, and integrated platforms. This is especially useful in heterogeneous tissues including the brain, retina, and skeletal muscle [69]. Additional mechanistic insight is provided by spatial transcriptomics, which further correlates changes in gene expression with particular tissue regions and microenvironments [70].

Network-based and machine-learning techniques, such as co-accessibility networks, expression quantitative trait loci (eQTL) mapping, and multi-omics factor analysis (MOFA), are used by integrating computational frameworks to interpret these complicated datasets. Information on post-translational modifications and subsequent impacts on protein abundance is added by incorporating proteomics [71].

Robust computing infrastructure, high-quality reference datasets, and strong cooperation between molecular scientists, bioinformaticians, and clinicians are necessary for the successful implementation of these multi-omics techniques [72]. Mechanistic discoveries are brought closer to clinical translation by these methods, which enhance diagnosis accuracy for rare illnesses and can find prospective treatment targets by exposing the influence of noncoding mutations on cellular pathways [73]. Representative experimental and computational approaches for noncoding variant discovery and validation are summarized in Table 2.

## 5. Case Studies of Selected Monogenic Diseases

Monogenic disorders offer intriguing case studies to comprehend the role of regulatory and noncoding variations in human disease. Although exonic mutations are still the most well-understood causes of monogenic conditions, recent WGS and transcriptome studies show that noncoding variants account for an estimated 10–20% of cases that remain unsolved after WES, highlighting their measurable contribution to the diagnostic gap [79]. By bridging genomic discovery and translational therapies, these case reports demonstrate how investigation of the noncoding genome has converted unresolved genetic diagnoses into mechanistically characterized illnesses. We examine five representative disorders below where the onset, severity, or responsiveness to treatment are considerably altered by deep intronic, promoter, enhancer, and UTR variations [80].

### 5.1. Cystic Fibrosis

Mutations in the CFTR gene, which encodes a chloride channel essential for electrolyte and fluid transport in organs such as the colon, pancreas, lungs, and sweat glands, cause cystic fibrosis (CF), a classic autosomal recessive disorder [81]. The ΔF508 coding mutation is the most prevalent of the more than 2000 CFTR variations that have been found. The significance of regulatory and noncoding systems in disease is illustrated by the fact that many clinically diagnosed CF patients have unresolved issues that cannot be resolved by traditional exome sequencing [82].

Aberrant mRNA transcripts and dysfunctional chloride channels can arise from deep intronic mutations that introduce pseudoexons or activate cryptic splice sites. A well-characterized example that encourages pseudoexon inclusion is c.3718-2477C>T (3849+10kbC>T). Exome sequencing usually misses these mutations, which can only be found by RNA-seq of patient-derived epithelial cells, WGS, or targeted intronic analysis [83].

By interfering with transcription factor binding and lowering transcriptional efficiency, changes to the CFTR promoters and enhancers may alter gene expression. These regulatory abnormalities may affect the severity of the disease, especially in patients who have the same coding mutation, even though they are less frequent than intronic mutations [84]. Differences in CFTR’s untranslated regions, such as the 5′ and 3′ UTRs, can affect microRNA binding, translation efficiency, and mRNA stability. Despite their incomplete characterization, these mutations are thought to change the levels of the CFTR protein and contribute to different phenotypic consequences [85].

Finding noncoding mutations in CFTR has important therapeutic implications. Expanded molecular testing has made it possible to definitively diagnose patients who were previously classified as “mutation-negative.” Mechanistic insights into deep intronic mutations have also driven the development of antisense oligonucleotides (ASOs), such as eluforsen (QR-010), which mask cryptic splice sites and restore normal splicing. These results show how identifying noncoding variations improves diagnostic performance and directs precision RNA-based treatments for cystic fibrosis [86].

### 5.2. β-Thalasemia (HBB)

Insufficient production of the β-globin chain leads to β-thalassemia, an autosomal recessive blood disorder that causes anemia with varying severity [87]. Although the HBB gene has several known coding mutations, regulatory and noncoding variants also play a major role in determining the disease’s symptoms, particularly in atypical or borderline cases. Indeed, long before genome-wide analyses were accessible, β-thalassemia was among the first examples of how regulatory mutations can result in Mendelian traits [88,89].

Promoter mutations such as −28A>G and −29A>G lead to reduced transcription of HBB and decreased production of β-globin by disrupting transcription factor binding at essential regulatory regions [35]. These variants demonstrate how single-nucleotide changes in noncoding regions can give rise to clinically meaningful phenotypes. Splicing mutations such as IVS-I-110 G>A and IVS-II-654 C>T lead to the formation of abnormal splice donor or acceptor sites, leading to aberrant mRNA and reduced β-globin synthesis. RNA studies on patient-derived samples have confirmed the detrimental effects of these mutations, demonstrating reduced amounts of β-globin and abnormal splicing patterns [24,90].

Although less common, variations in the untranslated regions of HBB can influence mRNA stability and translational efficiency. Minor variations in expression can influence disease severity, especially in compound heterozygotes [91]. Detecting noncoding variants is important for prenatal counseling and enhances the accuracy of carrier screening and molecular diagnostic procedures. Furthermore, the development of gene therapy and gene-editing techniques to reactivate fetal hemoglobin expression to compensate for inadequate β-globin synthesis has been guided by a better understanding of promoter and intronic abnormalities [92].

### 5.3. Duchenne Muscular Dystrophy

DMD is an X-linked condition caused by mutations in the DMD gene encoding for the structural protein dystrophin, which is necessary for maintaining muscle integrity. Dystrophin deficiency or malfunction leads to early mortality and progressive muscular deterioration. Noncoding variations have become significant contributors to DMD, although massive deletions and duplications are frequent [93,94].

Variants located deep within introns can create frameshifted transcripts, resulting in truncated dystrophin proteins through the inclusion of pseudoexons or activation of cryptic splice sites. Detection of these variants typically requires WGS or focused intronic analyses, as they are frequently overlooked by standard exome sequencing [95]. Less frequently, mutations in promoter or enhancer regions can modify the onset or severity of DMD by altering transcriptional activity. These effects have been investigated through functional investigations, including those that use luciferase reporter assays, CRISPR-mediated gene editing, and patient-derived myoblasts [96].

Since noncoding DMD variations increase eligibility for exon-skipping and splice-modulating RNA treatments, which restore the reading frame and allow the synthesis of partially functional dystrophin, their identification is therapeutically significant. Thorough variant finding improves genetic counseling, helps with patient stratification for clinical trials, and improves prognostic assessment [97,98].

### 5.4. Familial Hypercholesterolemia

Familial hypercholesterolemia (FH) is an autosomal dominant illness characterized by elevated LDL cholesterol, tendon xanthomas, and early-onset cardiovascular disease. The LDLR gene, which codes for the LDL receptor, is mutated in the majority of instances [99]. Regulatory and noncoding variants have occasionally been blamed for unresolved instances using traditional sequencing, despite the fact that coding mutations have been the subject of much research [100,101].

Promoter mutations that interfere with transcription factor binding, including changes in Sp1 motifs, can lower LDLR transcription and cause elevated cholesterol. Deep intronic variations can disrupt splicing, while enhancer mutations, though less frequent, can impact gene expression in a tissue-specific way [102,103].

Incorporating regulatory polymorphisms into diagnostic assessments significantly enhances variant detection in patients exhibiting distinct clinical symptoms despite the absence of coding mutations (13). Insights into the genetic consequences of promoter or enhancer variants directly impact therapeutic planning, genetic counseling, and assessment of heritable risk. Patients with regulatory variants may benefit from intensified lipid-lowering strategies such as statins, PCSK9 inhibitors, or novel RNA-based therapeutics that increase hepatic LDLR expression [104].

### 5.5. Inherited Retinal Disorders

Inherited retinal disorders (IRDs) represent a diverse group of genetic diseases that lead to gradual vision loss, sometimes progressing to total blindness. Although advances in exome sequencing have greatly improved diagnostic rates, 25–40% of IRD cases remain unsolved after WES, largely because of the wide range of genes and mechanisms involved [105,106].

Significant functional impacts could be exhibited by deep intronic variations. Notably, a cryptic splice site is produced by the CEP290 c.2991+1655A>G mutation, which leads to the inclusion of pseudoexons and the production of a non-functional protein [107]. Likewise, progressive retinal degeneration is linked to deep intronic mutations that impact splicing in ABCA4 and USH2A. Despite being very uncommon, promoter and enhancer mutations have a significant impact on gene expression, which in turn affects the start and course of the disease [108].

WGS allows for the identification of intronic and regulatory variations in people without a molecular diagnosis, hence these discoveries have significant ramifications [109]. Clinical research is also being conducted on targeted therapeutics; for instance, current clinical trials are being carried out to correct the inclusion of the CEP290 pseudoexon utilizing ASOs. In IRDs, thorough identification of noncoding variations promotes individualized treatment plans, supports patient classification for gene- and RNA-based therapeutics, and improves genetic counseling [110,111]. Representative examples of monogenic diseases where noncoding variants contribute to pathogenesis are summarized below in Table 3.

The pathophysiology and phenotypic diversity of many monogenic conditions have been shown to be significantly influenced by dysregulation of noncoding RNAs, including lncRNAs, miRNAs, and circular RNAs, in addition to promoter, enhancer, intronic, and UTR alterations [112]. CircRNAs act as miRNA sponges or regulators within ceRNA networks, lncRNAs affect chromatin accessibility and transcriptional regulation, and miRNAs control gene dosage, according to numerous studies. Emerging data indicate that ncRNA-mediated regulation frequently interacts with or amplifies the effects of underlying genomic variants, although these mechanisms are still being clarified for specific remain poorly understood in profiling monogenic diseases. This highlights the significance of incorporating ncRNA pro-filing into future diagnostic and therapeutic frameworks [113,114].

**Table 3:** Examples of monogenic diseases affected by noncoding variants, illustrating molecular mechanisms and clinical implications for diagnosis and therapy.

**Disease**	**Gene(s)**	**Variant Type**	**Molecular Mechanism**	**Clinical Relevance**
Cystic Fibrosis [27,83]	*CFTR*	Deep intronic, promoter/enhancer, UTR	Pseudoexon inclusion, altered transcription, disrupted mRNA stability	Improves molecular diagnosis Informs ASO therapy Precision modulators
β-Thalassemia [90]	*HBB*	Promoter, intronic, UTR	Reduced transcription, aberrant splicing, altered mRNA stability	Enhances genetic counseling, carrier screening, prenatal testing Guides HbF-based therapies
Duchenne Muscular Dystrophy [115]	*DMD*	Deep intronic, promoter/enhancer	Cryptic splice site activation, pseudoexon inclusion, reduced transcription	Expands eligibility for exon-skipping therapies Informs prognosis and clinical management
Familial Hypercholesterolemia [116,117]	*LDLR*	Promoter/enhancer, intronic	Reduced LDL receptor expression, altered splicing	Improves diagnosis in negative-coding cases Guides treatment intensity and family screening
Inherited Retinal Disorders [118]	*CEP290*, *ABCA4*, *USH2A*	Deep intronic, promoter/enhancer	Pseudoexon inclusion, aberrant splicing, altered transcription	Resolves previously unsolved cases Informs ASO and gene-based therapeutic strategies

## 6. Clinical Implications and Therapeutic Opportunities

### 6.1. Improved Molecular Diagnosis

The discovery of noncoding and regulatory variations has enhanced the molecular diagnosis of monogenic diseases. Many cases remain unresolved because standard WES frequently overlooks deep intronic, promoter, enhancer, and UTR variants. By combining transcriptome profiling and WGS, these hitherto unknown variations can be found and their functional relevance evaluated [119,120].

For example, deep intronic CFTR variants that introduce pseudoexons or disrupt normal splicing were later found to be present in a significant percentage of patients with suspected cystic fibrosis but negative WES results [28]. Similarly, noncoding regulatory mutations in HBB, DMD, CEP290, and LDLR have clarified unexplained phenotypes across multiple clinical contexts [95,121,122,123]. These findings demonstrate that going beyond the exome enhances diagnostic resolution and identifies previously unidentified mechanistic factors.

The early detection of noncoding variants supports timely diagnosis, individualized treatment, and more accurate genetic counseling, including carrier screening and reproductive planning [124]. Therefore, thorough assessments of regulatory and noncoding variations are necessary to close the ongoing “diagnostic gap” in rare disorders.

### 6.2. Therapeutic Relevance of Noncoding Variants

Novel targeted therapeutics have been made possible by a growing understanding of the molecular mechanisms behind noncoding variation. For instance, ASOs can correct deep intronic mutations that create pseudoexons in CFTR and CEP290, restoring proper splicing and functional protein expression [30,107]. Similarly, insights into intronic and splice-altering variants have enabled exon-skipping therapies in DMD, generating truncated yet functional dystrophin [115].

Despite being less frequent, promoter and enhancer deficits present chances for transcriptional modification by small compounds, gene therapy, or CRISPR-based tools. Technologies such as CRISPR activation (CRISPRa), CRISPR interference (CRISPRi), and epigenome editing can enhance or suppress gene expression and even correct aberrant histone marks or DNA methylation [125,126].

In addition to direct correction, knowledge of these regulatory systems helps with precision pharmacotherapy by enabling forecasts of the ways in which transcript abundance, splicing efficiency, and translation dynamics affect drug response. Overall, knowledge of the noncoding genome is influencing individualized treatment plans and stimulating novel therapeutic techniques [127,128].

### 6.3. Implications for Genetic Counseling and Prognosis

The prognosis of patients, risk assessment, and genetic counseling all depend on the identification of noncoding variations. By influencing the intensity of symptoms, explaining the variation in phenotypes across people with similar coding mutations, and displaying partial penetrance, polymorphisms might improve the precision of prognostic predictions [18,45]. For example, promoter variants in HBB or regulatory variants in CFTR might affect residual gene expression and hence modify clinical severity, whereas enhancer variants in BCL11A that raise fetal hemoglobin levels can significantly alter the severity of sickle cell disease and β-thalassemia. Despite having the same coding mutations, noncoding modifiers have also been demonstrated to accelerate or slow the course of several cardiomyopathies [129,130,131].

Preconception and preimplantation genetic testing greatly benefit from the more thorough assessment of inherited risk made possible by the addition of noncoding variant data. Molecular diagnostic tools now help clarify how regulatory variants influence gene expression, offering valuable guidance for both immediate and long-term care [132,133]. For example, understanding how a promoter variant reduces gene activity can inform whether a patient might benefit from RNA-based therapy or emerging gene-editing strategies such as CRISPR-Cas9. In practice, these insights allow clinicians to make more informed treatment choices rather than relying solely on phenotypic observation [134].

Moreover, when patients’ individual genetic profiles are taken into account, genetic counseling becomes more specific and actionable. For instance, someone with a variant linked to hereditary cardiomyopathy might receive early lifestyle recommendations or monitoring plans tailored to their risk. This personalized approach strengthens clinical decision-making and can meaningfully improve patient outcomes over time [135,136].

### 6.4. Future Integration in Clinical Practice

Recent advances in bioinformatics and genomics demonstrate that successfully integrating knowledge from several fields will be essential to converting findings about noncoding variants into medical application [137]. For instance, researchers have already identified disease-causing variations in illnesses like muscular dystrophy and specific neurodevelopmental disorders by combining WGS with RNA-seq. This type of integrated analysis enables the detection of both genomic changes and their subsequent impact on gene expression in diagnostic situations [138].

However, gathering data alone is insufficient. To maintain repeatability and clinical dependability, systematic processes for functional validation, variation classification, and prioritization must be established and standardized. For example, somewhat different procedures are frequently used by laboratories interpreting noncoding variations, which might result in contradictory findings across research [45,139]. Inadequate annotation of enhancers and promoters, uncertainty in computational estimates of variation impact, and the challenge of evaluating tissue-specific regulatory activity are other present constraints. These obstacles make it difficult to accurately determine the pathogenicity of several noncoding variations [89].

Identification of pathogenic regulatory variations can potentially be enhanced by computational integration of transcriptomic, epigenomic, and genomic datasets. To link certain noncoding mutations to alterations in tissue-specific gene expression, the ENCODE and GTEx databases provide an excellent illustration [140]. Accelerating translational research and fostering cooperation would be achieved by creating carefully vetted, publicly accessible libraries that connect verified noncoding variations to observed clinical outcomes [141].

Beyond monogenic variation, diagnostic procedures are progressively integrating new multigenic frameworks, including genome-wide risk models and polygenic risk scores (PRS). In people whose phenotypes cannot be entirely described by a single, high-impact mutation, these methods help improve risk interpretation by quantifying the cumulative effect of common variations [142,143]. Recent research shows that combining PRS with rare-variant analysis might improve diagnostic interpretation in unclear instances, improve condition classification, and shed light on intricate genomic architectures [144]. These integrative methods demonstrate the future possibility of merging rare and common variant signals to boost precision-medicine approaches, even though PRS is not currently regularly utilized in strictly monogenic diagnostics.

Ultimately, these advances need to be incorporated into decision-support systems that assist clinicians in diagnosis, prognosis, and treatment planning. Achieving this goal will require coordination between molecular biologists, geneticists, bioinformaticians, and healthcare providers. A key component of precision medicine, as these cooperative frameworks develop, will be the meticulous interpretation of noncoding variation, which will allow for more individualized, molecularly informed treatments for patients with uncommon or monogenic disorders [145,146].

## 7. Challenges and Future Directions

### 7.1. Challenges in Variant Interpretation

It is still challenging to determine the function of noncoding and regulatory variations in human genetics, despite advancements in sequencing and functional genomics. Noncoding variants affect transcription, splicing, and RNA stability in context-dependent ways, in contrast to coding mutations, which directly affect protein function [147,148]. Accurate predictions of functional or pathogenic consequences are hampered by the regulatory genome’s inadequate mapping, especially for long-range elements like enhancers and silencers [149].

Although machine learning technologies such as SpliceAI, DeepSEA, CADD, and EIGEN aid in variant selection, their efficacy varies depending on the tissue, stage of development, and experimental setup [150]. Depending on the cellular context, a variant’s influence changes since regulatory systems are frequently tissue-specific. VUSs are therefore still widely used, mostly in datasets that are produced from WES [151]. Furthermore, it is difficult to confirm pathogenicity because there are no standardized norms for regulatory variants that are similar to the ACMG/AMP guidelines for coding changes. This typically necessitates considerable experimental validation. It is still urgently necessary to establish consensus approaches for evaluating noncoding variations [45,152].

### 7.2. Technical Limitations

Despite WGS’s extensive coverage of noncoding areas, its diagnostic value is limited by a number of technological limitations. Complex insertions that upset regulatory architecture, structural rearrangements, and repetitive or GC-rich areas are difficult for short-read platforms to precisely resolve [48]. The resolution of repetitive sections, haplotype phasing, and the capture of full-length transcripts are all made possible by long-read sequencing technologies like Oxford Nanopore and PacBio HiFi, which can resolve many of these problems. Broader clinical implementation remains constrained by the need for specialized instrumentation, limited throughput, and substantial costs [153,154].

Although they have practical constraints, functional assays offer an additional crucial perspective on understanding variations. Although RNA-seq, CRISPR screens, and minigene testing might provide valuable information, they usually miss developmental or tissue-specific contexts [155]. Organoids and iPSCs are patient models that offer more realistic systems, although they are technically sophisticated and labor-intensive. Adaptive diagnostic methods must thus strike a compromise between clinical feasibility and in-depth variation analysis [156,157].

### 7.3. Integration of Multi-Omics Data

Multi-omics data integration is a useful method for resolving variation interpretation issues. Researchers can establish a strong link between changes in DNA sequence and subsequent biological effects by integrating genomic, transcriptomic, epigenomic, and proteomic data. They can therefore make a stronger argument for the possibility of being harmful [158].

Combining WGS with RNA-seq, ATAC-seq, and techniques like Hi-C and Capture-C that map the arrangement of chromatin is possible. This aids in the identification of changes that change the structure of the three-dimensional genome, chromatin accessibility, or gene expression [159,160]. Additionally, single-cell multi-omics can concurrently examine the quantities of proteins, RNA, and DNA accessibility in a single cell. The regulatory processes that are difficult to monitor in bulk studies are revealed in this way [161]. In complex tissues such as the brain, retina, and muscle, where diverse cell types and finely tuned regulation generate distinct molecular and functional profiles, these techniques prove especially useful [162].

The integration of diverse datasets introduces significant analytical and computational complexity, necessitating standardized analysis pipelines, uniform metadata structures, and interoperable data formats to ensure interpretability [163,164]. Furthermore, translating multi-omics discoveries into therapeutic applications will require accessible analytical methods and effective visualization tools. The continued progress of this field will depend on close collaboration among clinicians, computational biologists, and molecular scientist [165].

### 7.4. Future Directions

Advancing the therapeutic application of noncoding variant interpretation necessitates both rigorous experimental validation and robust computational prediction frameworks [45]. Deep learning architectures, including Basenji2 and Enformer, leverage raw DNA sequence information to model and predict tissue-specific gene expression profiles. While regulatory variant analysis has advanced significantly, existing models must undergo further development, comparative assessment, and validation to ensure dependable and interpretable clinical application [166].

Comprehensive databases that link clinical and phenotypic metadata to experimentally verified noncoding mutations would increase dependability and facilitate clinical translation. Systematic curation of regulatory variants in databases such as ClinVar and gnomAD is enabling greater diagnostic precision and improving the consistency of variant interpretation [167].

Ultimately, the progress of precision medicine relies on the integration of noncoding variant analysis into routine diagnostics and therapeutic development. Clinical practice is changing as a result of developments in functional testing, computer modeling, and sequencing technologies. It is anticipated that applying these techniques to the regulatory genome will enhance risk prediction, resolve diagnostic problems, and provide patients with monogenic illnesses with additional therapeutic options [168,169].

## 8. Conclusions

There is growing recognition of the significance of noncoding and regulatory variants in monogenic disorders. Changes in deep intronic regions, UTRs, promoters and enhancers, and noncoding RNAs can explain conditions that exome sequencing cannot resolve. Through the use of transcriptomics, functional testing, WGS, and computational predictions, researchers have improved CRISPR- or RNA-based treatments, exon-skipping, splice-switching ASOs, genotype-phenotype connections, and variant finding.

Despite these achievements, there are still many obstacles to overcome in order to identify pathogenic variants, validate their functional significance, and translate findings into effective treatments. Future studies should focus on developing integrated variant databases, expanding the use of high-throughput functional tests, and enhancing predictive models in order to provide patients with monogenic illnesses with more accurate diagnoses and customized treatments.

## Figures and Tables

**Table 1:** Summary of major categories of noncoding variants and their mechanisms in monogenic diseases, with representative gene examples and clinical relevance.

Variant Category	Location	Molecular Mechanism	Example Genes	Notes
Promoter/Enhancer Variants [38]	Upstream regulatory regions (promoters, enhancers)	Altered transcription-factor binding Disrupted enhancer–promoter interaction leading to abnormal transcription	*HBB*, *LDLR*, *PAX6*, *SOX10*	Modulates gene expression and influences phenotype severity
Deep Intronic Variants [28]	Intronic regions far from canonical splice sites	Cryptic splice-site activation Pseudoexon inclusion causing aberrant splicing	*CFTR*, *CEP290*, *DMD*	Often missed by WES; detected by WGS or targeted intronic assays
5′/3′ UTR Variants [39]	Untranslated regions	Altered mRNA stability or translation Disrupted miRNA binding	*FMR1*, *GATA2*, *MECP2*	Contributes to phenotype variability via post-transcriptional regulation
Noncoding RNA Variants (lncRNA, miRNA, circRNA) [40]	lncRNA loci, miRNA genes, circRNA back-splice junctions	Disrupted regulatory networks Altered miRNA sponging; ceRNA imbalance	*ANRIL*, *BGas* (CFTR), miR-144/451, circRNAs in DMD/IRDs	Rapidly expanding evidence for contribution to monogenic diseases

**Table 2:** Summary of genomic, transcriptomic, and functional approaches used to detect and interpret noncoding variants, with representative applications in monogenic disease research.

Method	Purpose	Key Features	Examples/Applications
WGS [74]	Variant discovery	Covers coding and noncoding regions; long-read sequencing captures complex variants	Detection of deep intronic CFTR or CEP290 variants
RNA-seq [75]	Assess transcript consequences	Detects aberrant splicing, altered transcript abundance	Confirmation of pseudoexon inclusion in *CFTR*, *CEP290*
Minigene Reporter Assays [27]	Functional validation	Tests specific variant effect on splicing	Validating intronic or UTR variants
CRISPR-based Screens [76]	Functional genomics	Target promoters, enhancers, or noncoding RNAs	Identifying regulatory elements in *DMD*, *LDLR*
Computational Prediction Tools [77]	Prioritize candidates	SpliceAI, DeepSEA, CADD, EIGEN	Predict pathogenic potential of noncoding variants
Multi-Omics Integration [78]	Comprehensive interpretation	Combines genomics, transcriptomics, epigenomics	Links variants to functional consequences and clinical relevance

## Data Availability

No new data were created or analyzed in this study. Data sharing is not applicable to this article.
